# Harm reduction-the cannabis paradox

**DOI:** 10.1186/1477-7517-2-17

**Published:** 2005-09-22

**Authors:** Robert Melamede

**Affiliations:** 1 Biology Department, 1420 Austin Bluffs Parkway, University of Colorado, Colorado Springs, 80918, USA; 2 Bioenergetics Institute, 1420 Austin Bluffs Parkway, University of Colorado, Colorado Springs, 80918, USA

## Abstract

This article examines harm reduction from a novel perspective. Its central thesis is that harm reduction is not only a social concept, but also a biological one. More specifically, evolution does not make moral distinctions in the selection process, but utilizes a cannabis-based approach to harm reduction in order to promote survival of the fittest. Evidence will be provided from peer-reviewed scientific literature that supports the hypothesis that humans, and all animals, make and use internally produced cannabis-like products (endocannabinoids) as part of the evolutionary harm reduction program. More specifically, endocannabinoids homeostatically regulate all body systems (cardiovascular, digestive, endocrine, excretory, immune, nervous, musculo-skeletal, reproductive). Therefore, the health of each individual is dependant on this system working appropriately.

## Introduction

The concept of harm reduction is at the heart of conflicting international drug policies. The Dutch pioneered this approach. Today most European countries and Canada have embraced the idea that society benefits most when drug policy is designed to help people with drug problems to live better lives rather than to punish them. In contrast, the United States federal policy demands rigid zero tolerance with overwhelming emphasis on incarceration of offenders (the Drug War). Although, seemingly reasonable arguments can be made to support both sides of the dispute, the recent global trend towards harm reduction has resulted from the acknowledgement that drug use has been a part of all societies throughout history and the realization that repressive policies are expensive, ineffective, and often harmful.

A dramatic example of the benefits that can result from a harm reduction approach to drugs is seen with needle exchange programs. While prohibitionists argue that providing clean injection equipment promotes drug use, the facts do not support this contention. For example, the Australian needle exchange program is credited with keeping the HIV/AIDS infection rate very much lower than what is typically found globally . Commonly cited examples of the failed repressive policies championed by the United States are the now repealed alcohol prohibition and the current drug war. Crime, financial support for terrorism, disrespect for the law, and destruction of families, communities, and ecosystems can all be attributed to drug prohibition. Yet, the staggering cost of the drug war, driven by United States policy and taxpayers' money, amounts to many billions of dollars a year.

Cannabis is the third most commonly used drug in the world, following tobacco and alcohol. In the United States, much of the drug war is focused on marijuana (over 700,000 people arrested last year alone). Is there justification for this policy? The gateway theory states marijuana use leads to the use of other drugs, and drives the U.S. policy despite evidence that suggests alcohol and tobacco use may foster the gateway effect [1,2]. In contrast, countries that support harm reduction focus their enforcement and social support efforts on "hard drugs." Consequently, many countries have effectively decriminalized marijuana. Holland, having the most liberalized drug laws, does not have more cannabis users (over age twelve) than do more repressive countries, and the per capita number of heroin users is also lower . The Dutch Ministry of Justice estimates that 0.16% of cannabis users are heroin users. This figure does not support cannabis being a gateway drug. Data from the 2000 National Household Survey on Drug Abuse (U.S. Department of Health and Human Services, Substance Abuse and Mental Health Services Administration) also shows that the vast majority of people who try cannabis do not go on to use hard drugs.

A little explored question is what does harm reduction specifically mean with respect to cannabis consumption? This article will address cannabis harm reduction from a biological perspective. Two directions will be examined: what are the biological effects of cannabis use and what are the social effects that emerge from the biological foundation.

Like many substances that are put into the human body, there can be positive or negative consequences that result from cannabis consumption, depending on amount, frequency, quality, and probably most importantly, the idiosyncratic biochemistry of the user. Prohibitionists concentrate their efforts on the negative effects of cannabis use, while anti-prohibitionists tend to focus on the positive effects. If we assume that both sides have valid arguments, the issue to be resolved is one of balance between the negative and positive effects. Would a policy of tolerance, or prohibition, be more likely to reduce harm overall? Which policy would better serve society as a whole, as well as problematic drug users?

Biological science can be more objectively evaluated than social science. The central theme that will be presented in this article is that appropriate cannabis use reduces biological harm caused by biochemical imbalances, particularly those that increase in frequency with age. Proper cannabis use, as distinguished from misuse, may have significant positive health effects associated with the way cannabis mimics natural cannabinoids. In essence, it is proposed that the endocannabinoid system, selected by 600 million years of evolution, is a central mediator of biological harm reduction through its homeostatic activities. The social implications of cannabis use will be viewed as emerging from the biological platform. Herein lies the paradox of cannabis and harm reduction. Is appropriate use of cannabis better than no use?

## The Controversy

Cannabis use can be divided into three categories, recreational, medical, and religious. The latter will not be examined in this article. Some, including those who favor or oppose cannabis use, presume recreational and medical use are the same. On the one side, it is often claimed that any cannabis use is justified by some underlying medical need. On the other side, cannabis use is presumed to have no medical value, with the implication that those who use it are simply "getting stoned." While the former claim may be too extreme, the latter defies current scientific understanding of the biological functions of the endocannabinoids. While many people are reluctant to approve recreational cannabis use, it appears that most people support medical use. The United States Federal Government denies that there is any valid medical use for cannabis, while the National Institute of Drug Abuse (NIDA) provides marijuana on a monthly basis to a few medical users through the compassionate Investigatory New Drug (IND) program of the Food and Drug Administration (FDA). Nevertheless, a number of states, through either legislative action or voter initiative, have approved the use of medical marijuana[3].

## Current Federally Approved Medical Marijuana Uses

In order to better assess arguments for and against the medical use of marijuana, the scientific evidence for the health benefits of cannabis will be reviewed below. It should be noted that the federally supplied cannabis users have been receiving and using cannabis for 11 to 27 years with clinically demonstrated effectiveness in the treatment of glaucoma, chronic musculoskeletal pain, spasm and nausea, and spasticity of multiple sclerosis [4]. Furthermore, there is no evidence that these patients have suffered any negative side effects from their cannabis use.

## The Endocannabinoid System

Cannabis preparations have been used medically for thousands of years for illnesses such as epilepsy, migraine headaches, childbirth, and menstrual symptoms. However, it is only relatively recently that the active components have been identified and their mechanisms of action have begun to be understood. While delta-9-tetrahydrocannabinol (THC) was first synthesized by Mechoulam in 1967 [5], it was not until 1990 that the cannabinoid receptor was localized in the brain [6] and cloned [7]. Since then, discoveries in the field have proceeded at an ever-increasing pace. The discovery of cannabinoid receptors on cells naturally prompted the search for internal compounds (endogenous ligands) that would activate the receptors since it seemed unlikely that cannabis receptors had evolved so people could partake of cannabis. In 1992, anandamide was discovered [8]. This lipid metabolite was the first ligand of an ever-expanding class of molecules known as endocannabinoids (internal marijuana-like compounds) to be discovered. Endocannabinoid synthesis, degradation, transport, and receptors together form the endocannabinoid system.

The broad therapeutic potential that can result from correctly manipulating the endocannabinoid system is just beginning to be realized[9,10]. In fact, major pharmaceutical companies, and university researchers all around the world are now engaged in the cannabinoid-related research [11]. Their efforts focus on learning how the endocannabinoid system functions, and on how to manipulate it in order to increase or decrease its activity, depending on the illness or condition under consideration. GW Pharmaceuticals in Britain has been developing and testing a plant extract-based product line that is in clinical trials in Britain and Canada [12]. The results thus far have been positive to the extent that Bayer AG has entered into a 25-million-dollar distribution agreement for GW's product, Sativex which has recently been approved in Canada. In contrast, Sanofi Research has developed an antagonist that will inhibit the ability of endocannabinoids to stimulate hunger and thus potentially be useful for weight control.

## Evolution of Endocannabinoids

The cannabinoid system appears to be quite ancient [13,14], with some of its components dating back about 600 million years to when the first multicellular organisms appeared. The beginnings of the modern cannabinoid system are found in mollusks [15] and hydra [16]. As evolution proceeded, the role that the cannabinoid system played in animal life continuously increased. It is now known that this system maintains homeostasis within and across the organizational scales of all animals. Within a cell, cannabinoids control basic metabolic processes such as glucose metabolism [17]. Cannabinoids regulate intercellular communication, especially in the immune [18] and nervous systems [19]. In general, cannabinoids modulate and coordinate tissues, organ and body systems (including the cardiovascular [20], digestive [16], endocrine [21], excretory [22,23], immune [18], musculo-skeletal [24], nervous [19], reproductive [25], and respiratory [26] systems). The effects of cannabinoids on consciousness are not well understood, but are well known, and underlie recreational cannabis use. These effects also have therapeutic possibilities [27].

## Cannabinoids: Homeostatic Regulators

The homeostatic action of cannabinoids on so many physiological structures and processes is the basis for the hypothesis that the endocannabinoid system is nothing less than a naturally evolved harm reduction system. Endocannabinoids protect by fine-tuning and regulating dynamic biochemical steady states within the ranges required for healthy biological function. The endocannabinoid system itself appears to be up- or down-regulated as a function of need. As will be detailed later in this article, endocannabinoid levels naturally increase in the case of head injury and stroke [28], and the number of cannabinoid receptors increases in response to nerve injury and the associated pain [29]. In contrast, the number of cannabinoid receptors is reduced when tolerance to cannabinoids is induced [30].

## Physical Characteristics of Living Systems

To illustrate the multidimensional biochemical balancing act performed by cannabinoids, a variety of endo- and exocannabinoid activities will be reviewed below. In order to appreciate these activities a brief introduction to cell biology may provide the context for this review. All life is dependant upon the maintenance of its dynamic organization through sufficient input of nutrients and removal of wastes. The more complicated an organism is, the more complex the coordination required to accomplish the essential tasks necessary to maintain this vital flow of inputs and outputs. Coordination requires communication. Cells communicate by thousands of different, but specific, receptors on cell surfaces that respond to thousands of different, but also specific, molecules (ligands) that bind to the receptors. A receptor that is bound to its activating ligand causes biochemical changes to occur in the cell. In response to such regulatory signals on the membrane, biochemical regulation within the cell occurs at the level of gene expression as well as at the level of enzyme action and other processes outside the nucleus. Ultimately these changes, through complex biochemical pathways, allow cells to divide, carry out specialized tasks, lie dormant, or die. Any of these cellular activities, when not properly coordinated, can result in illness. Two major categories of disease states are those that result from acute illness commonly caused by infections and those that are age-related. Historically, in the United States, the cause of death has transitioned from being pathogen-induced to age-related. Current scientific literature regarding cannabis indicates that its use is often bad for the former but good for the latter (see Immunology section below).

## Cannabinoids and Brain Disorders

Since cannabis' action on the brain is most widely known due to its recreational use, the nervous system will serve as the starting point for examining cannabinoid activity as an example of a natural biological harm reduction system. Numerous disease states associated with the nervous system will be seen as potential targets for cannabinoid-based therapy [31]. The nervous system is composed of nerve and supporting cells. In addition to the role cannabinoids play in a healthy nervous system [32], the regulatory effects of cannabinoids in cases of stroke [28], Parkinson's disease [33], Huntington's disease [34], amyotrophic lateral sclerosis (ALS) [35], Alzheimer's disease [36], glioma (a type of brain tumor), [37] multiple sclerosis [38], seizures[39], and pain [40,41] will be examined.

## Cannabinoids and the Healthy Brain

In a healthy individual, cannabinoids play a direct role in neurotransmission of many nerve cell types. They exhibit the unusual property of retrograde transmission, in which the cannabinoid neurotransmitter diffuses backwards across the neural cleft to inhibit the presynaptic action potential [42]. This function essentially regulates the sensitivity of a nerve cell by acting as a feedback mechanism that prevents excessive activity. Some nerve cells die when they are excessively stimulated by excitatory neurotransmitters (excitotoxins) such as glutamate. Cannabinoids can reduce the level of stimulation and protect against this form of cell death [43,44]. In addition to their down-regulatory effect on neurotransmission, cannabinoids play other roles in reducing this type of cell death (biological harm reduction) by regulating the role of interleukin-1 (IL-1, an inflammatory cytokine) and the IL-1 receptor antagonist (IL-1ra) [45]. For example, cannabinoids were shown to modulate the release of IL-1ra thereby protecting against IL-1 assisted cell death [46].

The role of cannabinoids in neurological health and disease goes beyond the prevention of cell death and regulates neuronal differentiation. Cannabinoid receptors are functionally coupled to the fibroblast growth factor receptor (FGF). The FGF receptor, when stimulated, activates lipid catabolism via diacylglerol (DAG) lipase which causes the hydrolysis of DAG to produce 2-arachidonyl glycerol (2AG) [47]. 2AG is an endocannabinoid shown to be important for axon growth and guidance[48]. This function is critical for nerves to innervate their target effectors. The ability to control these fundamental neurological activities, in conjunction with the anti-inflammatory properties of cannabinoids, is likely to have important regenerative health benefits for people suffering from neurological damage as occurs with stroke or injury [28].

## Multiple Sclerosis

Both animal and human studies provide strong evidence of the therapeutic potential of cannabinoids to provide relief from a number of neurological disease states [49]. The use of cannabinoids to treat people suffering from multiple sclerosis (MS) is an excellent example of the importance of "medical marijuana" as an agent of harm reduction[50] MS is a neurodegenerative disease in which the immune system attacks components of the nervous system. The axons of many central nervous system (CNS) neurons are surrounded by a myelin sheath that acts much like an insulator around a wire. MS is associated with the degradation of the myelin sheath that leads to loss of axon function and cell death, thus producing the disease symptoms.

Cannabis-based therapies for the treatment of MS can provide symptomatic and true therapeutic relief. On the one hand, cannabinoids help to reduce spasticity in an animal model of MS (chronic relapsing experimental autoimmune encephalomyelitis (CREAE) [51]. However, the involvement of the cannabinoid system in the etiology of MS goes much deeper. MS is in reality an autoimmune disease. In order to appreciate why cannabinoids can have in important role, beyond what has already been mentioned, in treating MS on a mechanistic level [52], a brief introduction to immunology is required.

## Cannabinoids and the Immune System

The role of the immune system is simplistically thought of as protecting us from foreign attack. More inclusively, however, the immune system has the biological function of modulating the life, death, and differentiation of cells in order to protect us. The immune system accomplishes these tasks, in part, by balancing two mutually opposed pathways known, respectively, as the "Th1" and "Th2" response. The Th1 immune response is critical for fighting infections caused by specific infectious agents [53]. This function is inhibited by cannabinoids. Thus cannabinoids are important homeostatic modulators of the immune system. While often classified as immune inhibitors, cannabinoids actually promote the Th2 response while they inhibit the Th1 response. Therefore cannabinoids are immune system modulators. A specific cannabinoid receptor (Cb2) [54] is found on most cells of the immune system.

## Th1 Immune Response

The Th1 pathway is proinflammatory and functions by inducing the defensive production of free radicals that are vital for fending off pathogens, especially intracellular pathogens, such as those that cause Legionnaire's disease, Leishmania, and tuberculosis. Accordingly, the use of cannabis should be avoided when the Th1 arm of the immune system is needed to fight a particular disease. Although contagion as well as immune suppression may have been involved, a recent study supports this perspective, in that a cluster of new tuberculosis cases was traced to a shared water pipe [55]. Free radical production, inflammation and cell-mediated immunity are characteristic of the Th1 response. The targeting of infectious organisms, or infected cells, by a Th1 immune response results in healthy surrounding cells being exposed to free radicals. Much as if radiation had been applied, there is collateral damage that occurs with a targeted Th1 immune response.

## Cannabinoids and Th1 Mediated Auto-Immune Diseases

In contrast to the Th1 immune response, the Th2 immune response promotes the humoral arm of the immune system. It turns down the Th1 response, is characterized by antibody production, and is typically anti-inflammatory. Ideally, the Th1 and Th2 pathways are functionally balanced to optimally meet the survival needs of an organism in its environment. In reality however, many autoimmune diseases, and other age related diseases, are characterized by an excessive Th1-driven immune response at the site of the of the tissue damage involved. Multiple sclerosis, arthritis, Crohn's disease, and diabetes are all diseases that fall into this category.

The therapeutic impact of cannabinoids on these diseases can be dramatic. For example, when rodents were given experimental autoimmune encephalomyelitis (EAE) as an MS animal model and were treated with cannabinoids, the results were profound [56]. In a study that involved both guinea pigs and rats, 98% of the EAE animals that were not treated with THC died. In contrast, greater than 95% of THC-treated animals survived. They had only mild symptoms with a delayed onset or no symptoms at all. The capacity of cannabinoids to down-regulate a spectrum of auto-immune diseases should serve as a warning against the long term use of CB1 inhibitors for weight control. Such drugs are currently in the regulatory pipeline [57] and one of the participants in the clinical trial unexpectedly developed multiple sclerosis [58].

## Cannabinoid Actions-Biphasic Responses

The brief interludes into cell biology, neurology, and immunology provide a biological platform for considering how cannabinoids might impact a variety of other disease states. It is important to keep in mind that in its role as a general homeostatic modulator, too much or too little cannabinoid activity can be harmful. Cannabinoid levels or concentration ranges vary as a function of an organism's genetics, the cell types under consideration, and their health and environment. Care must be taken when evaluating the scientific literature on cannabinoids and their effects. Cannabinoids often exhibit biphasic responses [59]. Low doses of cannabinoids may stimulate the Th2 immunological response, whereas high doses may inhibit the Th2 response and shift the balance in favor of a Th1 response. From a harm reduction perspective, these observations demonstrate the critical importance of dose-dependent, disease-dependent, state-dependent, and individually tailored approaches to cannabis therapeutics [60].

The use of cannabinoids in the treatment of Parkinson's disease is an example of a condition where excessive or deficient cannabinoid activity may prove problematic. Parkinson's disease results from the loss of levo-dopamine (L-dopa) producing neurons. In an animal model of Parkinson's disease, L-dopa producing cells are killed with 6-hydroxydopamine. Rats so treated exhibit spontaneous glutamatergic activity that can be suppressed by exo- as well as endocannabinoids [61]. The standard treatment for Parkinson's disease involves L-dopa replacement therapy. Unfortunately, this treatment often results in dyskinesia (abnormal voluntary movements). Recent clinical trials have shown that cannabinoid treatment reduces the reuptake of gamma-aminobutyric acid (GABA) and relieves the L-dopa-induced dyskinesia [33], as well as L-dopa induced rotations in 6-hydroxydopamine-lesioned rats [62]. In contrast to the potential benefits of cannabinoid agonists just cited, using a different animal model, the cannabis antagonist SR141716A reduced reserpine-induced suppression of locomotion [63]. Thus, in this model locomotion was restored by inhibiting the endocannabinoid pathway.

## Cannabinoids and Cancer

Possibly the greatest harm-reducing potential afforded by cannabinoids comes from their use by cancer patients. Cannabinoids possess numerous pharmacological properties that are often beneficial to cancer patients. Many people are aware of the anti-emetic and appetite stimulating effects of cannabinoids [64]. A systemic study designed to quantify the efficacy of cannabinoids as an anti-emetic agent examined data from 30 randomized controlled studies that were published between 1975 and 1997 and included 1366 patients who were administered non-smoked cannabis [65]. For patients requiring a medium level of control, cannabinoids were the preferred treatment (between 38% and 90%). This preference was lost for patients requiring a low or a high level of control. Sedation and euphoria were noted as beneficial side effects, whereas dizziness, dysphoria, hallucinations, and arterial hypotension were identified as harmful side effects.

The cancer cell killing [66] and pain relieving properties of cannabinoids are less well known to the general public. Cannabinoids may prove to be useful chemotherapeutic agents [67]. Numerous cancer types are killed in cell cultures and in animals by cannabinoids. For example, cannabinoids kill the cancer cells of various lymphoblastic malignancies such as leukemia and lymphoma [68], skin cancer [69], glioma [70], breast and prostate cancer [71], pheochromocytoma [72], thyroid cancer [73], and colorectal cancer[74]. Since 2002 THC has been used in a clinical trial in Spain for the treatment of glioma [75]. However, not all cancers are the same, and cannabinoid-induced biochemical modifications, while effective in killing the cells of some cancers, as indicated above, can have the opposite effect on the cells of other types of cancer. For example, recent work has shown that the synthetic cannabinoid, methanandamide, can promote the growth of lung cancer cells by a receptor independent pathway that involves the up-regulation of COX2 [76]. Although much has been learned about the therapeutic value of cannabinoid agonists and antagonists in different situations, scientific understanding of how to appropriately modulate the endocannabinoid pathways remains preliminary, with much remaining to be learned.

## Cannabinoids and Pain

One area of current research that has begun attracting public interest is the pain relieving potential of cannabinoids, for both cancer [77] and non-cancer patients [78]. Medicine based on cannabis extract has demonstrated positive effects for pain relief [79]. Recently, an intrinsic role for cannabinoids in pain circuitry was discovered: the endocannabinoid AEA was identified as the natural ligand for the vanilloid receptors [80]. Vanilloid receptors, which are ligand-gated cation channels, are primary targets for the treatment of pain [81]. The cannabinoids seem to function in a pathway parallel to the opioid pathway [82] and are thought to exert anti-nociceptic activity at the level of the spinal cord and the brain [83], although they can also act peripherally by inhibiting mast cell degranulation [84]. In recognition of the pain relieving properties of cannabinoids, England [11] and Canada [41] are using cannabis preparations to provide relief to citizens suffering from a variety of disorders. Human trials have established that co-administration of cannabinoids can dramatically lower opioid use and can provide pain relief for neurogenic symptoms where other treatments have failed [85]. Recently, the topical application of the synthetic cannabinoid WIN 55,212-2 significantly enhanced the antinociceptive activity of morphine, opening the door for possible cannabis-induced pain relief with reduced cognitive side effects [86]. The intrinsic role of endocannabinoids in modulating pain is further supported by the up-regulation of the CB1 receptor in rats following nerve damage [29]. Once again, nature has selected cannabinoids to reduce harm.

## Smoking and Lung Cancer

Fundamental to any consideration of cannabis-based harm reduction, as a biological phenomenon or as a policy, is how to best administer the drug. Smoking cannabis preparations, in contrast to oral administration [87], has the benefit of rapid action that allows self-titration of the drug's activity [88,89]. Unfortunately, cannabis smoke contains numerous carcinogenic compounds [90]. In fact, cannabis smoke may contain more tars than tobacco smoke [91]. However, despite the fact that cannabis smoke does produce cellular changes that are viewed as precancerous, a major epidemiological study does not find that cannabis smoking is associated with tobacco related cancers [92]. A number of recent studies provide a scientific foundation for the clear relationship between tobacco smoking and lung cancer, a relationship that does not hold true for cannabis smoke (manuscript submitted to HRJ). For example nicotine, acting via nicotine receptors, is critical in the development of tobacco related cancer by inhibiting the death of genetically damaged cells [93]. Tobacco also promotes the development of blood vessels needed to support tumor growth [94] whereas cannabis inhibits tumor vascularization in nonmelanoma skin cancer [69] and glioma [95]. Although conclusions derived from an oft-cited study examining the carcinogenic effects of cannabis, tobacco, and cannabis combined with tobacco claims to show a link between cannabis smoking and head and neck cancer [96]. But these results do not hold up under scrutiny. The study does support a link between tobacco use that is exacerbated by concurrent cannabis use and the development of head and neck cancer. However, the "cannabis use only" group was composed only of two subjects, undermining the statistical relevance of conclusions regarding this group.

## Smoking Alternatives

Regardless of whether or not smoking cannabis can cause lung cancer, smoking anything containing partially oxidized hydrocarbons, carcinogens, and irritants a priori, is not healthy and will have negative health consequences. Fortunately, harm-reducing alternatives exist. While often touted as a problem, the availability of high THC cannabis with high levels of THC permits less cannabis to be smoked for therapeutic effects. Additionally, methods of vaporizing the active ingredients of cannabis have been shown to successfully remove most compounds of concern while efficiently delivering the desired ones [97]. These results contrast with a recent Australian study that found that the use of a water pipe, or bong, failed to reduce tars or carbon monoxide delivered to the smoker [98]. GW Pharmaceuticals is developing an oral spray that should prove to be an additional safe and effective alternative delivery system [12] and valuable to medical cannabis users. The company has also identified strains with defined ratios of various cannabinoids for which specific medicinal value will be determined.

## Cannabinoids Affect Drug Metabolism

Another important cannabis and harm reduction topic that must be considered is that of how the use of cannabis impacts on the pharmacokinetics of other drugs [99]. A number of drugs are metabolized by the P450 family of isoenzymes, including numerous cannabinoids [100]. Even though cannabinoids stimulate the transcription of P450 (2A and 3C), they also directly inhibit the activity of this enzyme [101]. There are likely to be pros and cons associated with P450 inhibition. P450 activity activates procarcinogens in tobacco smoke to create active cancer-causing mutations [102]. Thus, the inhibition of these enzymes by cannabinoids may minimize some of the negative consequences of smoke inhalation. On the other hand, many pharmaceutical drugs are metabolized by these enzymes. The reduction of the rate of drug metabolism by cannabinoids with pharmokinetic consequences has been shown for cocaine [103], barbiturates [104], opiates [105], alcohol, the antipsychotic haloperidol [106], and others [107].

Thus far, both endo- and exocannabinoids are seen to reduce harm in numerous circumstances. Cannabinoid-based therapies have been especially helpful for the treatment of a variety of neurological and immunological disorders. Yet, we have only scratched the surface of the scientific literature on cannabinoids and their biological effects. Nevertheless, it should be apparent that cannabinoids have enormous medical potential as we learn to manipulate the natural cannabinoid harm reduction system that has evolved in the animal kingdom.

A fundamental question that remains unanswered is how basic, complex biochemical phenomena, as touched on briefly in this article, collectively emerge as substantial contributors to health and behavior. In far-from-equilibrium thermodynamic systems, such as living organisms, there are discontinuities between underlying molecular dynamics and associated emergent macroscopic phenomena [108]. In such systems, small changes (called "perturbations") can amplify with consequences for the organization of the whole system. The cannabinoids help to regulate an amazingly broad range of biochemical events. All of these effects have genetic foundations. As such, natural genetic/biochemical variation in a population can be expected to have significant effects on health and behavior. It should be expected that in a population distribution of cannabinoid levels and sensitivities, as a function of an individual's health/disease status, some individuals would naturally need to increase their cannabinoid activity while others would need theirs lowered. Although the focus of this paper has been to suggest the many circumstances in which higher cannabinoid activity would be beneficial, these circumstances will necessarily differ among individuals with different congenital cannabinoid levels and sensitivities. Therefore, reduced cannabinoid activity would be beneficial under some conditions. A prime example of potential harmful effects of excess cannabinoids is their effects on pregnancy where low levels are needed but high levels are harmful [109].

## Behavioral Effects: Self-administration and Reward

The broad homeostatic activities of cannabinoids that have been developed in this article have been rooted in hard science. The extension of these ideas to the psychological and behavioral levels is intrinsically more speculative, but remains consistent with the literature. For years, researchers have looked into the possible addictive qualities of cannabis. The lack of significant reward behavior was indicated by the lack of self-administration in primates. Experiments examining preference in rats demonstrated that low doses of THC could induce place preference but that higher doses produced drug aversion [110], again demonstrating the homeostatic nature of cannabinoids. Self-administration is typical of most psychoactive drugs of abuse. Hence, one could conclude that marijuana has a low potential for abuse.

Some may question the conclusion that cannabis has a low abuse potential since an animal model using squirrel monkeys was recently developed in which self-administration behavior was maintained using THC [111]. Interestingly, and consistent with the notion that the cannabinoid system is a biological homeostatic harm reduction mechanism, the self-administration of THC ranges from 2 to 8 ug/kg and peaks at 4 ug/kg [112]. Thus, in this animal model a controlled dose is chosen. To further put these experiments in perspective, the dose used must be examined more closely. A 1-gram joint of 10% THC content would contain 100 mg of THC. The self-administered dose schedule chosen by the animal of 4 ug/kg would correspond to 360 ug of THC (if absorption was complete, approximately 1/278 of the joint) for a 200-pound human. Similarly, in rats, the intravenous self-administration of the synthetic cannabinoid Win 55,212-2 also occurred in a biphasic manner, with a maximum response occurring at 12 ug/kg[113] The self-regulated, controlled use of low drug doses is not characteristic of addictive drugs of abuse.

Additional cannabinoid involvement in reward behavior is suggested by the increased activity of dopaminergic neurons stimulated with psychoactive cannabinoids [114]. This pathway is shared by other major drugs of abuse including, morphine, ethanol, and nicotine [115]. However, the production of glucocorticoid hormones that are normally produced in response to stress [116], are suppressed by cannabinoids [117]. Are cannabinoids addictive, is pleasure addictive, or is a low stress state addictive?

## Cannabinoids and Stress

Stress and reward are complicated components of addictive behavior. How does repeated use of THC influence these states? A recent study examines this question by measuring glucose utilization in different areas of the rat brain following repeated treatment with THC [118]. After 7 and 21 days of THC treatment, THC no longer resulted in reduced glucose utilization in many areas of the brain typically affected by a single THC dose (most cortical, thalamic, and basal ganglia regions). In contrast, glucose utilization in other areas of the brain remained unaltered (nucleus accumbens, mediodorsal thalamus, basolateral amygdala, portions of the hippocampus and median raphe). Thus while the effects of THC on body temperature and locomotor activity become resistant to repeated THC administration, those areas involved in many higher brain functions remain responsive to THC. This differential adaptation to THC administration is consistent with a low addictive potential. The best evidence that demonstrates the absence of an addictive response to cannabis use is the fact that most people who use it do not continue to use it, and stop using it without any effort.

The stress-relieving properties of cannabinoids are an important aspect of their pharmacological activity. An interesting mechanism by which cannabinoids may promote stress relief is through their effects on memory. Cannabinoids control the extinction of painful memories [119]. What a blessing for those suffering from debilitating or life threatening illnesses: cannabinoids may help them to forget their misfortune.

Independent of the direct addictive or non-addictive properties of cannabis, the cannabis-opioid connection will be examined in more detail. Both drug families function (not necessarily exclusively) through biochemical pathways that are regulated by specific receptor-ligand interactions. However, there appears to be, as yet not fully defined, crosstalk between these pathways [120]. For example, CB1 receptor knockout mice are non-responsive to CB1 cannabinoid activities and show reduced addictive effects of opiates [121]. Similarly, Lewis rats showed enhanced sensitivity to morphine self-administration after treatment with the synthetic cannabinoid CP55040 [122]. Examining the cannabis-opioid connection from the other direction, chronic morphine administration results in some down-regulation of cannabinoid receptors along with a significant reduction in 2AG [123]. These results show both positive and negative feedback relationships between the endocannabinoid and opiate systems. They also suggest that cannabinoids might serve to reduce the symptoms of opiate withdrawal [124].

The possibility that cannabinoids could serve as an addiction interrupter was demonstrated in rats where the synthetic cannabinoid agonist Win 55-212,2 reduced intravenous self-administration of cocaine [125]. Similarly, recent studies indicate that THC may facilitate nicotine withdrawal in mice [126] and inhibit alcohol preference in a model of alcoholism [127]. The opposite indications, that blocking cannabinoids receptors could serve as an addition interrupter has also been made [128].

## Behavioral Complexity

Behavioral processes and their complexities set humans apart from other animals. Can we simply extrapolate from animal to human behavior? It is one thing to comparatively examine the molecular and cell biology of animals and extrapolate to humans. However, the behavioral repertoire of humans appears to be dramatically enhanced over other animals and is therefore more difficult to connect between the species. Evolutionary relationships show that the cannabinoid receptors are located in the more advanced areas of our brains. Again, any population is always a spread around the average value of any parameter. A subset of the human population will inevitably retain a more primitive behavioral repertoire. Is this subset more susceptible to addictive behavior or psychological problems that could result from cannabis consumption? Has the cannabinoid system been optimized for the regulation of more primitive behavior or, alternatively, is it better optimized for the behavioral flexibility required of modern humans? Indeed, is there any evidence that the cannabinoid system, like our cortical capacity, may enable even greater behavioral flexibility in the more complex societies and altered environments of the future?

Answers to these questions are suggested by the data of human cannabis consumption. Most people who use cannabis in their youth stop using it as their lives progress. Most do so as a natural part of their development. They do so without outside intervention or help. They do so without ever having become heroin users, schizophrenic, or motivationally compromised. These facts indicate that for the majority of people who try marijuana, it is not addictive, does not lead to heroin use, nor is it a trigger for the onset of psychological problems. However, due to the complexity of cannabinoid activities, it is likely that in a small percentage of the population, cannabis use may foster problems. The biology presented in this paper suggests that such individual differences should be expected. We must learn to identify individuals who would be negatively affected by cannabis use; they are the people that an intelligent drug policy would help to identify and assist. In contrast, our policy criminalizes the majority of users and further harms them, perhaps psychologically as well as medically, through its repercussions.

The use of cannabis – and any mind-altering drug – by young developing minds rightfully remains an area of focus and concern. For example, is there a relationship between cannabis use and schizophrenia? Schizophrenia is characterized by distortions of reality, disturbances of language and thought processes, and social withdrawal. Certainly, aspects of cannabis intoxication parallel these symptoms. It is feared that cannabis can precipitate this state [129], especially in susceptible individuals [130]. It has been suggested that schizophrenics (or potential schizophrenics) fall into two categories with respect to cannabis use [131]. One group may find symptomatic relief in the use of cannabis, while the other may actually take the risk of inducing the onset of the disease. The complexities of this issue are illuminated by the unpredictable behavior of interacting complex systems such as the nervous and immune systems, as will be considered below.

In an important recent study, De Marchi et al [132], examined the endocannabinoid levels in healthy volunteers and compared them to that of schizophrenic patients, both before and after successful antipsychotic treatment. Patients suffering with acute disease had significantly higher anandamide levels in their blood than did the normal individuals or patients in clinical remission. Might these elevated cannabinoid levels be contributing to the disease symptoms, and what might be causing them? Cannabinoids act homeostatically across biological subsystems. A possible immune involvement in schizophrenia has long been suspected, and immunological parameters have been implicated in the disease. For example, there is an inverse correlation between schizophrenia and rheumatoid arthritis; an individual generally does not get both illnesses [133]. Interestingly, schizophrenia has been correlated with HLA type, *Toxoplasma gonodii *infection, and exposure to cats [133]. *Toxoplasma gonodii *infects brain neurons, and is best controlled with a strong pro-inflammatory immune response. Endocannabinoids modulate the pro-inflammatory TH1 response by up-regulating the anti-inflammatory Th2 response. Hence, it is likely that some individuals idiosyncratically respond to *Toxoplasma gonodii *infections by producing excess endocannabinoids and suffering the associated abnormal mental state. Antipsychotic drugs have actually improved the outcome of infection with this parasite[134].

## Conclusion

Evolution has selected the endocannabinoids to homeostatically regulate numerous biological phenomena that can be found in every organized system in the body, and to counteract biochemical imbalances that are characteristic of numerous damaged or diseased states, in particular those associated with aging. Starting from birth, cannabinoids are present in mother's milk [135], where they initiate the eating process. If the activity of endocannabinoids in the mouse milk is inhibited with a cannabinoid antagonist, the newborn mice die of starvation. As life proceeds, endocannabinoids continuously regulate appetite, body temperature, reproductive activity, and learning capacity. When a body is physically damaged, the endocannabinoids are called on to reduce inflammation, protect neurons [136], regulate cardiac rhythms [137] and protect the heart form oxygen deprivation [20]. In humans suffering from colorectal cancer, endocannabinoid levels are elevated in an effort to control the cancer [74]. They help relieve emotional suffering by reducing pain and facilitating movement beyond the fears of unpleasant memories [119].

While this review is far from complete, it attempts to provide a conceptual overview that supports the endocannabinoid system as being nature's method of harm reduction. There is a pattern to all the cannabinoid-mediated activities described. Many of the biochemical imbalances that cannabinoids protect against are associated with aging. Aging itself is a system-wide movement towards chemical equilibrium (away from the highly regulated far-from-equilibrium state) and as such is an imbalance from which all living organisms suffer. In contrast, the harmful consequences of cannabis use, however exaggerated they often appear to be, are likely to represent significant potential risk for a minority of the population for whom reduced cannabinoid levels might promote mental stability, fertility or more regulated food consumption.

## Supplementary Material

Additional File 1 It contains hyperlinks to the text document. Click here for file
