# Supplementary material for: Harm reduction-the cannabis paradox
Source: Harm Reduct J. 2005 Sep 22;2:17. doi: 10.1186/1477-7517-2-17 (PMC1261530; doi:10.1186/1477-7517-2-17)
Supplement: Additional File 1 — It contains hyperlinks to the text document. [file 1477-7517-2-17-S1.doc]

**Pro Links**

Pg 2 The concept of harm reduction http://www.thebody.com/encyclo/harm_reduction.html

Pg 2 the Drug War http://en.wikipedia.org/wiki/War_on_Drugs

Pg 2 needle exchange programs http://en.wikipedia.org/wiki/Needle_exchange_program

Pg 2 the facts do not support this contention http://www.hhs.gov/news/press/1998pres/980420a.html

Pg 2 financial support for terrorism http://www-hoover.stanford.edu/pubaffairs/we/2002/henderson_0502.html

Pg 2 destruction of families http://www.lewrockwell.com/miller-joel/miller-j3.html

Pg 2 ecosystems http://www.drugpolicy.org/drugwar/environment/index.cfm

Pg 2 over 700,000 people arrested last year alone http://www.fbi.gov/ucr/03cius.htm

Pg 2 the most liberalized drug laws http://www.cedro-uva.org/lib/reinarman.dutch.html

Pg 2 2000 National Household Survey on Drug Abuse http://www.health.org/govstudy/nhsda2000/

Pg 3 appropriate cannabis use reduces biological harm caused by biochemical imbalances http://www.rxcbc.org/exj.html

Pg 3 the way cannabis mimics http://www.ccguide.org.uk/brainche.html

Pg 3 most people support medical use http://www.bostonphoenix.com/boston/news_features/editorial/documents/01636588.htm

Pg 3 a number of states, through either legislative action or voter initiative, have approved the use of medical marijuana http://www.cannabisnews.com/news/thread19794.shtml

Pg 4 Cannabis preparations have been used medically for thousands of years http://www.skunked.co.uk/articles/marijuana-history.htm

Pg 5 These effects also have therapeutic possibilities http://www.bma.org.uk/ap.nsf/Content/drugTherapeuticusePU

Pg 5 good for the latter http://www.davidhadorn.com/cannabis/guidelines.htm#elderly

Pg 6 The use of cannabinoids to treat people suffering from multiple sclerosis (MS) http://www.druglibrary.org/schaffer/hemp/medical/ms1.htm

Pg 8 Care must be taken when evaluating the scientific literature on cannabinoids and their effects http://www.cfdp.ca/canrep/Part_3-Chap_1.htm

Pg 9 use by cancer patients http://www.drugpolicy.org/marijuana/medical/challenges/litigators/medical/conditions/cancer.cfm

Pg 9 how to best administer the drug http://www.ukcia.org/medical/gwpharmaceuticals.html

Pg 10 harm reducing alternatives exist http://www.alternativesmagazine.com/31/bayer.html

Pg 11 cannabis has a low abuse potential http://www.drugscience.org/pfa/pfa_abuse.htm

Pg 13 Most people who use cannabis in their youth stop using it as their lives progress. http://www.cfdp.ca/lancet2.htm

Pg 13 without every having become heroin users http://www.dailyfreepress.com/news/2002/12/09/News/Marijuana.Not.A.Gateway.Drug.National.Study.Says-340102.shtml

Pg 13 schizophrenic http://www.msnbc.msn.com/id/5758223/

Pg 13 motivationally compromised http://www.erowid.org/plants/cannabis/cannabis_myth17.shtml

**Con Links**

p.3 health effects http://www.usdoj.gov/dea/ongoing/marijuanap.html

p.5 receptor http://www.thc.nl/Documents/BoRMarijuanaMayAffectEmbryo.htm

p.6 anti-inflammatory http://www.drugscope.org.uk/news_item.asp?a=1&intID=334

spasticity http://www.nationalmssociety.org/Sourcebook-Marijuana.asp

p.7 diabetes http://www.diabetesireland.ie/view.asp?ID=997

p.8 impact http://www.medicalpost.com/mpcontent/article.jsp?content=/content/EXTRACT/RAWART/3707/59A.html

p.10 cannabis smoking http://www.marijuanaaddiction.info/news-left.htm?aid=57

cannabis smoke http://www.nida.nih.gov/Infofax/marijuana.html

failed to reduce tars or carbon monoxide http://my.marijuana.com/pipestudy.php3

activates procarcinogens http://ajrcmb.atsjournals.org/cgi/content/full/24/3/339

p.11 abuse http://www.drugabuse.gov/MedAdv/99/NR-420.html

p.12 Stress and reward http://165.112.78.61/pubs/teaching/Teaching5.html

p.13 does not lead to heroin use http://www.marijuanaaddiction.info/marijuana-gateway-drug.htm

schizophrenia http://news.bbc.co.uk/1/hi/health/2407027.stm

p.14 symptoms http://www.sciencedaily.com/releases/2004/06/040615075809.htm

precipitate http://www.pubmedcentral.nih.gov/articlerender.fcgi?artid=135490&rendertype=abstract

take the risk http://www.psychosocial.com/addiction/gersabec.html

p.15 harmful consequences http://www.usdoj.gov/dea/pubs/cngrtest/ct961202.htm

**Omni Links**

p. 1 Robert Melamede, Ph.D. http://www.uccs.edu/~biology/faculty/melamede.htm

p. 3 natural cannabinoids

http://www.lacbc.org/science.html

p. 4 delta-9-tetrahydrocannabinol (THC)

http://www.hempreport.com/issues/17/malbody17.html#Cannabinoids%20and%2

0THC

p. 4 anandamide

http://c4.cabrillo.cc.ca.us/features/news.html#1

p. 5 molecules (ligands)

http://www.rism.org/isg/dlp/ganja/analyses/Marijuana%20and%20Medicine%202

a.html#endog

p. 6 nerve cell

http://users.rcn.com/jkimball.ma.ultranet/BiologyPages/N/Neurons.html

p. 6 fibroblast growth factor receptor (FGF)

http://jorde-lab.genetics.utah.edu/people/reha/Reha.html

p. 6 multiple sclerosis (MS)

http://www.mult-sclerosis.org/whatisms.html

p. 7 immune system

http://www-micro.msb.le.ac.uk/MBChB/2b.html

p. 7 Legionella’s disease

http://www.hse.gov.uk/legionnaires/

p. 7 Leishmania

http://www.cdc.gov/ncidod/dpd/parasites/leishmania/factsht_leishmania.htm

p. 7 tuberculosis

http://www.cdc.gov/nchstp/tb/faqs/qa_introduction.htm#Intro1

p. 8 Parkinson’s disease

http://www.parkinsonsinfo.com/about_parkinsons/whathappens.html

p. 9 methanandamide

http://www.caymanchem.com/neptune/servlet/neptune/catalog/90070/a/z/template

/Product.vm

p. 10 oral spray

http://www.gwpharm.com/research_drug_delivery.asp

p. 12 dopaminergic neurons

http://www.sigmaaldrich.com/Area_of_Interest/Life_Science/Cell_Signaling/Key

_Resources/Pathway_Slides__Charts/Drug_Activation.html

p. 13 schizophrenia

http://www.schizophrenia.com/family/sz.overview.htm

p. 14 *Toxoplasma gondii*

http://mt.essortment.com/whatistoxoplas_rhjv.htm
